# Effects of photobiomodulation therapy on secondary healing of the palatal donor site after free gingival graft harvesting: a systematic review and meta-analysis

**DOI:** 10.1007/s10103-026-05073-2

**Published:** 2026-11-07

**Authors:** Huong Chu Thi Quynh, Tung Kien Hoang, Minh Huyen Dam, Duc Manh Nguyen Van, Linh Chi Pham

**Affiliations:** 1 Faculty of Dentistry, University of Medicine and Pharmacy, Vietnam National University, Hanoi, Vietnam; 2 Department of Prosthodontics, University of Medicine and Pharmacy, Vietnam National University, Hanoi, Vietnam; 3 Department of Prosthodontics, National Hospital of Odonto-stomatology, Hanoi, Vietnam

**Keywords:** Connective tissue harvesting, Free gingival graft, Low-level laser therapy, Palatal donor, Pain, Wound healing

## Abstract

This systematic review and meta-analysis evaluated photobiomodulation (PBM) therapy for postoperative pain and secondary wound healing at the palatal donor site after connective tissue harvesting. Following PRISMA guidelines, five databases were searched through 20 October 2025 for randomized controlled trials comparing PBM therapy with appropriate controls. Primary outcomes were postoperative pain (visual analogue scale, VAS) and epithelialization; secondary outcomes were analgesic consumption, remaining wound area, and bleeding. Random-effects meta-analysis was used, risk of bias was assessed with the Cochrane Risk of Bias 2.0 tool and certainty of evidence with GRADE. Nine randomized controlled trials with 299 participants were included. Day-1 pain could not be pooled. No statistically significant difference in postoperative pain was observed at day 3 or day 7, with reductions of approximately 1.2 and 0.4 points on a 0–10 scale. Complete epithelialization at 14 days was more than twice as frequent, with no significant difference at day 21. The remaining wound area at day 7 was smaller by approximately 22 mm². Heterogeneity was moderate for pain and low for healing outcomes. PBM therapy may increase the proportion of palatal donor sites achieving complete epithelialization at 14 days. For postoperative pain the evidence is very uncertain, and no statistically significant reduction was observed at either pooled time point. Most trials carried a high or unclear risk of bias, and treatment protocols were heterogeneous. Larger trials with standardized dosimetry are needed to establish the clinical value of PBM therapy at the palatal donor site.

**Supplementary Information:**

The online version contains supplementary material available at https://doi.org/10.1007/s10103-026-05073-2.

## Introduction

 An adequate width of keratinized gingiva is important for the functional and esthetic stability of the periodontium, and its absence is a common indication for soft tissue grafting [1, 2]. Two related autogenous techniques are used at the recipient site: free gingival grafting (FGG), in which a keratinized epithelialized graft is transplanted directly, and connective tissue grafting, in which the epithelial layer of a similarly harvested graft is removed before transplantation to improve esthetic outcomes and root coverage [2, 3]. Both techniques require harvesting autogenous tissue, typically from the hard palate, and both achieve high rates of clinical success at the recipient site [2, 3]. This success is frequently offset by postoperative morbidity at the palatal donor site, which remains a major barrier to patient acceptance [2, 4].

Donor site morbidity after palatal harvesting comprises several distinct clinical problems: postoperative pain, delayed re-epithelialization of the secondary intention wound, and, less frequently, excessive intraoperative or early postoperative bleeding [5]. Pain is the most consistently reported of these: patients frequently experience substantial discomfort in the days following surgery, which reduces oral health–related quality of life [4, 5]. A range of adjunctive interventions has been proposed to reduce these problems, including hemostatic agents, protective stents and dressings, herbal preparations, platelet-rich fibrin formulations, and photobiomodulation (PBM) therapy, also referred to in earlier literature as low-level laser therapy [4–6].

Despite this range of interventions, no protocol has been established that reliably accelerates wound healing while providing adequate analgesia without pharmacological side effects [4]. PBM therapy has been evaluated in several randomized controlled trials as an adjunct following palatal graft harvesting, with individual trials reporting reductions in postoperative pain and improvements in wound healing compared with control groups [6, 7]. These trials differ substantially in laser wavelength, energy density, application protocol, and outcome definitions, and their findings on pain relief conflict [6, 7]. No single trial can therefore establish the direction or magnitude of the effect, whereas pooling comparable outcomes across randomized comparisons can estimate both and quantify the heterogeneity between them. The aim of this systematic review and meta-analysis was therefore to evaluate the effect of PBM therapy as an adjunct for reducing postoperative pain and accelerating wound healing at the palatal donor site, and to characterize the heterogeneity and certainty of the available evidence.

## Materials and methods

### Protocol and registration

This systematic review and meta-analysis was conducted in accordance with Cochrane Collaboration guidance and is reported according to the Preferred Reporting Items for Systematic Reviews and Meta-Analyses (PRISMA) statement [8, 9]. The review protocol was registered in the International Prospective Register of Systematic Reviews (PROSPERO), registration number CRD420251162104.

### The focused question

The search strategy was developed based on the PICO question: “What is the efficacy of low-level laser therapy used on the secondary healing at the palatal donor area after connective tissue graft harvesting on patient experience and wound healing outcomes?”

### Eligibility criteria

Inclusion criteria were as follows. (1) Participants: patients undergoing free gingival graft or de-epithelialized connective tissue graft procedures resulting in secondary intention healing at the palatal donor site. (2) Intervention: PBM therapy applied to the palatal donor site for wound healing and symptom relief. (3) Comparison: a concurrent control group receiving either sham (placebo) laser irradiation or no photobiomodulation, with or without standard donor site management (sutures, gauze, an acetate palatal plate, a palatal acrylic stent, a periodontal dressing, or a haemostatic sponge or matrix). (4) Outcomes: pain, assessed with a visual analogue scale (VAS), and epithelialization as primary outcomes; wound remaining area, analgesic intake, and bleeding as secondary outcomes. (5) Study design: randomized controlled trials.

Exclusion criteria were: (1) studies in which the laser application under evaluation was ablative or thermal rather than photobiomodulatory; (2) studies in which the intervention and outcomes were evaluated only at the recipient site; (3) studies involving donor sites outside the palate; (4) studies involving oral surgical procedures other than free gingival or connective tissue grafts; (5) studies involving oral surgical procedures resulting in primary wound healing; and (6) studies with insufficient data for analysis.

### Search strategy

Two reviewers conducted an independent search of five electronic databases — PubMed/MEDLINE, EMBASE, Cochrane Central Register of Controlled Trials (CENTRAL), Scopus, and Web of Science — from inception to 20 October 2025. Only reports published in English were considered. No grey literature sources, trial registries or reference lists were searched. The search queries are presented in Appendix 1.

### Study selection

Two independent reviewers screened the initial records based on titles and abstracts. Citations that met the inclusion criteria or presented ambiguities were advanced to full-text assessment. Articles meeting all eligibility criteria were included in the review, while reasons for exclusion were documented. Discrepancies between reviewers were resolved through discussion, with any persistent conflicts settled by a third reviewer.

### Risk of bias assessment

To appraise the internal validity of the selected trials, their methodological quality was assessed using the Cochrane Risk of Bias 2.0 (RoB 2) tool, as recommended by the Cochrane Handbook for Systematic Reviews of Interventions [10]. Two independent reviewers evaluated each study, classifying the risk of bias as ‘low risk,’ ‘some concerns,’ or ‘high risk.’

### Data collection

Two independent, calibrated reviewers extracted data in parallel using a pre-tested spreadsheet. Extracted information comprised study design, number of participants, demographics, and surgical details. Specific laser parameters including wavelength, energy density, output power, duration, and session frequency were recorded. Outcomes (pain scores, analgesic intake, bleeding, and re-epithelialization rates), summary of results, and published journal names were also documented. Extracted values were checked against the original publications, and any discrepancies were resolved through consensus discussion. Where a group-level value required for pooling was not reported numerically in a source publication, it was derived from the corresponding published figure by visual digitization.

### Data synthesis

Statistical analyses were performed in R version 4.5.2 with the meta package (version 8.2.1). Mean differences (MD) and risk ratios (RR) with 95% confidence intervals (CI) were calculated for continuous and dichotomous outcomes, respectively. A random-effects model with inverse-variance weighting, with the between-study variance estimated by restricted maximum likelihood, was applied because of the expected clinical and methodological heterogeneity in photobiomodulation parameters, application protocols, and adjunctive analgesics. Heterogeneity was assessed using the I² statistic and categorized as negligible (0–25%), low (25–50%), moderate (50–75%), or high (75–100%). Statistical significance was set at *p* < 0.05. For continuous outcomes the standard deviations were used as reported by the trials. In a sensitivity analysis the standard deviations of the split-mouth trials were rescaled for within-patient correlations imputed at 0.3, 0.5, 0.7 and 0.9. The certainty of evidence for the primary outcomes was rated using the Grading of Recommendations, Assessment, Development and Evaluations (GRADE) approach across the domains of risk of bias, inconsistency, indirectness, imprecision, and publication bias. A 95% prediction interval was calculated for each pooled continuous outcome to describe the range of effects expected in a future trial. Where only two trials contributed to a time point and their estimates were inconsistent, the individual results are reported without pooling. Funnel plots and formal tests for small-study effects were not performed because fewer than ten trials contributed to any single analysis.

## Results

### Study selection

**Fig. 1:**
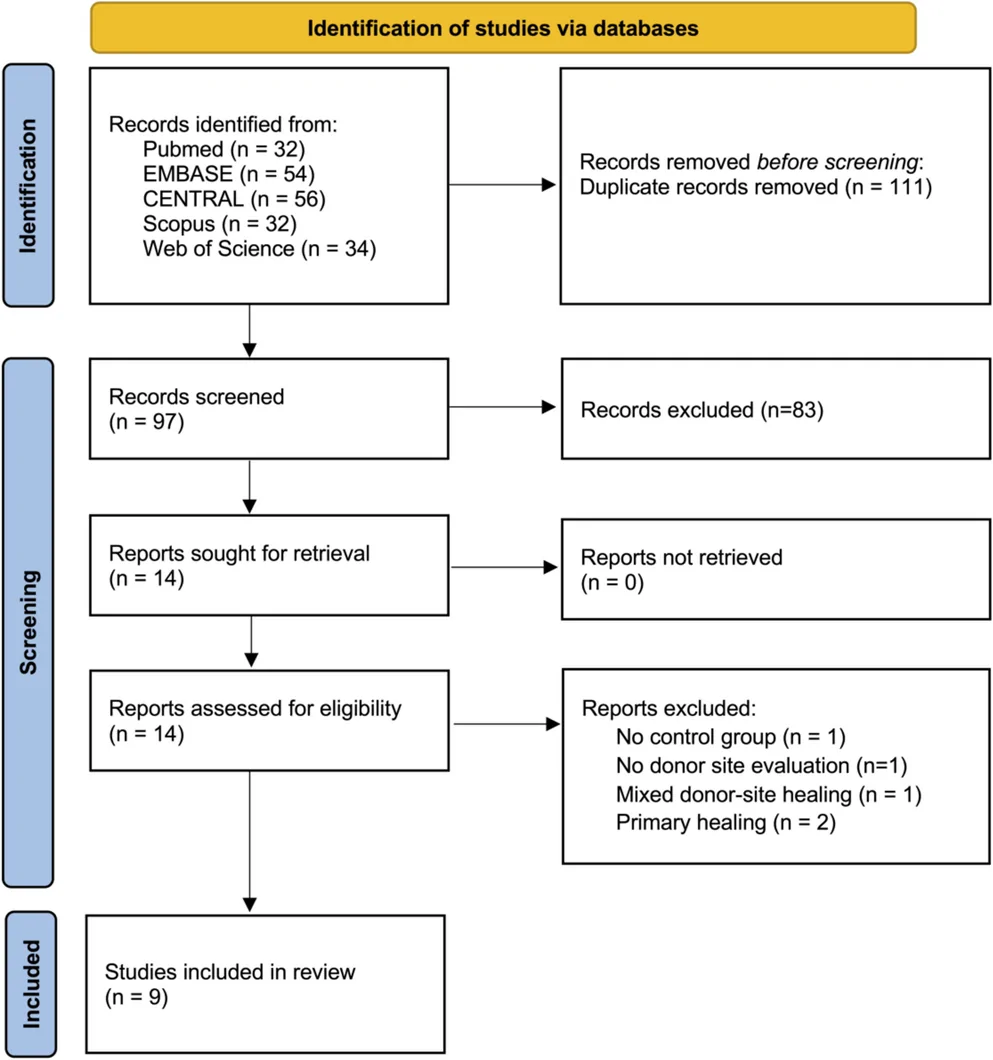
Flowchart of the study selection with reasons for exclusion

The database search identified 208 records from PubMed (*n* = 32), EMBASE (*n* = 54), CENTRAL (*n* = 56), Scopus (*n* = 32), and Web of Science (*n* = 34). Following duplicate removal (*n* = 111), 97 records underwent title and abstract screening, resulting in the exclusion of 83 irrelevant studies. All 14 potentially eligible full-text articles were retrieved and assessed against the predetermined inclusion criteria. One study was excluded because of the lack of a control group, two because they exhibited primary rather than secondary intention healing at the palatal donor site, one because it combined free gingival and connective tissue graft donor sites and did not report the donor-site outcomes separately, and one because the laser and placebo interventions and the reported outcomes were evaluated at the recipient site rather than at the palatal donor site. Nine studies met all eligibility criteria. The selection process is presented in Fig. 1.

### Study characteristics

The characteristics of the nine included trials are summarized in Table 1. Seven used a parallel-arm design and two a split-mouth design, and the number of participants analysed ranged from 12 to 52. The donor site tissue was harvested using the free gingival graft technique in all included trials; in several trials the harvested graft was subsequently de-epithelialized, either intraorally with a laser or extraorally with a blade, to produce a connective tissue graft for use at the recipient site.

**Table 1:** Characteristics of included studies. Variable parameters and outcomes of studies included in this systematic review (study design, participant numbers, surgical technique, laser type and parameters, postoperative analgesics, graft size, evaluated outcomes, summary of results, and journal)

Author et al. (year)	Study design	Participants *n* (C/T)	Surgery	Type of laser/wavelength (nm)	Output power	Energy density	Frequency of application	Duration
Bozkurt et al. (2025)	Parallel	36 (12/12/12)	FGG	GaAlAs/940	0.1 W	5 J/cm²	Immediately after surgery and at 3, 5, 7, 14 d later	30 s
Bastos et al. (2026)	Parallel	40 (20/20)	FGG	GaAlAs/660	0.1 W	1 J/cm²	Immediately after surgery and at 24, 48, and 72 h	20 s
Paz et al. (2025)	Parallel	30 (15/15)	DE-FGG	660/808	0.2 W	317.99 J/cm²	Immediately after surgery and 3, 7, 10, 15 d later	45 s (5 s each point)
Morshedzadeh et al. (2022)	Split-mouth	14 patients (28 sites; 14/14)	FGG	GaAlAs/940	0.21 W	5 J/cm²	Immediately after surgery, then every other day for 10 d (6 times)	30 s
Bitencourt et al. (2022)	Parallel	44 (22/22)	FGG	GaAlAs/660/808	0.1 W	107.14 J/cm²	Immediately after surgery and at 24 and 48 h later	30 s
Isler et al. (2018)	Parallel	36 (12/12/12)	FGG	Laser diode/970 ± 15	2 W	35 J/cm²	Immediately after surgery and 1, 3, 7 d later	30 s (6 s each point)
Ustaoglu et al. (2017)	Parallel	35 (18/17)	FGG	GaAlAs/940	3 W	34.4 J/cm² cumulative (8.6 J/cm² per session × 4 sessions)	Immediately after surgery and 3, 5, 7 d later	8 s each treatment
Heidari et al. (2017)	Split-mouth	12 patients (24 sites; 12/12)	FGG	Laser diode/660	0.2 W	32 J/cm²	Immediately after surgery and 1, 2, 4, 7 d later	32 s
Ozcelik et al. (2016)	Parallel	52 (26/26)	DGG	GaAlAs/810	0.1 W	4 J/cm²	Immediately after surgery	5 min
Author et al. (year)	Postoperative analgesics	Graft size	Outcomes	Summary of outcome	Journal			
Bozkurt et al. (2025)	Paracetamol	1.5–2 mm thick; area templated to recipient site	Pain (VAS 0–10), epithelialization, wound remaining area, analgesic consumption, bleeding	The total wound surface area on days 3, 7, 14, and 21 was significantly greater in the control group than in the test groups. VAS pain scores on days 3 and 4 were significantly higher in the control group. Analgesic consumption in the control group was higher than in the PBM group on postoperative day 3.	Clinical Oral Investigations			
Bastos et al. (2026)	Paracetamol (rescue)	1.5–2 mm thick	Epithelialization, pain (VAS; range not stated)	PBM therapy significantly reduced postoperative pain at the donor site compared with placebo at every time point from 3 h onwards; the two groups did not differ at 1 h. Participants receiving PBM therapy showed faster clinical healing, with a significant improvement observed 14 days after surgery.	Journal of Periodontal Research			
Paz et al. (2025)	Ibuprofen	–	Epithelialization, pain (VAS 0–10), wound remaining area, analgesic consumption	Dual-wavelength PBM therapy did not significantly influence overall wound closure, pain perception, or analgesic intake. Accelerated epithelialization was observed in the PBM group, with a higher proportion of donor sites exhibiting complete epithelial coverage by day 10 post-surgery.	Lasers in Medical Science			
Morshedzadeh et al. (2022)	Gelofen	9 × 14 × (1–1.5) mm	Epithelialization, pain (VAS 0–10), wound remaining area, color matching, bleeding	The test group achieved significantly smaller wound areas on days 7 and 14, although higher initial bleeding was experienced on the day of surgery compared with the control group. No significant differences were found between the groups regarding pain levels, discomfort, or long-term color match scores.	BMC Oral Health			
Bitencourt et al. (2022)	Ibuprofen (rescue use), acetaminophen	Minimum size of 12 × 7 mm	Pain (VAS 0–10), analgesic consumption, percentage of wound closure, patient-reported outcomes including bleeding	The PBM group required significantly fewer rescue analgesics and demonstrated superior recovery in daily functions, oral mobility, and symptom management (less swelling and bleeding) during the first 48 h. Faster wound closure was achieved by day 7, with no reported adverse effects.	Clinical Oral Implants Research			
Isler et al. (2018)	Flurbiprofen	9 × 11 mm	Epithelialization, pain and discomfort (VAS 0–10), wound remaining area, analgesic consumption, bleeding	At day 14, digital analysis showed significantly smaller wounds in the ozone group compared with the control, although clinician-based H₂O₂ evaluations found no significant difference. Postoperative pain did not differ significantly between groups at any time point, whereas by day 7 both the ozone and laser groups reported significantly less discomfort than the control group.	Photomedicine and Laser Surgery			
Ustaoglu et al. (2017)	Paracetamol	7 × 12 × ~1.5 mm	Epithelialization, pain (VAS 0–100), analgesic consumption, colour matching, bleeding, wound-healing index, tissue thickness	The PBM group demonstrated superior clinical recovery, characterized by higher epithelialization rates at day 14, reduced bleeding during the first 48 h, and consistently better wound healing index scores throughout the study. The control-group epithelialization rate at day 14 was derived from the published figure, as it was not reported numerically.	Photomedicine and Laser Surgery			
Heidari et al. (2017)	Gelofen	20 × 10 × 1 mm	Epithelialization, pain (VAS 0–100), analgesic consumption, bleeding	At day 14 and day 21, the difference between the two groups in complete epithelialization was statistically significant. During the first 3 h after surgery the mean pain value was significantly greater in the test group; from day 1 to day 12, no significant difference was noted between the treatment groups.	Journal of Photochemistry & Photobiology, B: Biology			
Ozcelik et al. (2016)	None prescribed	–	Discomfort (VAS; 10 cm line, no numerical anchors), tissue thickness	Statistically significant differences were found for oral health-related quality of life (*p* = 0.0001) and VAS (*p* = 0.0001) at the 7th postoperative day favoring test sites. Root coverage results did not show a statistically significant difference.	Journal of Clinical Periodontology			

*n: *number, *T:* test group,* C:* control group,* FGG:* free gingival grafting,* GaAlAs:* gallium-aluminum-arsenide,* PBM: *photobiomodulation,* VAS:* visual analogue scale, *d*: day, *hr*: hour

Most trials used diode lasers, including gallium-aluminum-arsenide (GaAlAs) devices. Reported single wavelengths varied across trials: 660 nm (*n* = 2), 810 nm (*n* = 1), 940 nm (*n* = 3), and 970 nm (*n* = 1). Two further investigations employed dual-wavelength protocols combining 660 nm and 808 nm simultaneously. Energy densities ranged from 1 to 317.99 J/cm². In every trial the first irradiation was delivered immediately after surgery, and most trials added further sessions, giving between one and six applications in total over periods of up to 15 days after surgery. In most trials the control site received a sham irradiation. Donor site management in the control arms ranged from no dressing or stent at all to sutures, an acetate palatal plate, a palatal acrylic stent, a periodontal dressing, a haemostatic sponge, or a collagen matrix (Table 2). Clinical follow-up assessments extended from 14 days to 6 months to evaluate postoperative morbidity and tissue healing (Table 1).

**Table 2:** Donor site management in each study arm and study-specific definition of complete epithelialization

Author et al. (year)	Donor site management, experimental arm	Donor site management, control arm	Assessment method and definition of complete epithelialization
Bozkurt et al. (2025)	Periodontal dressing (Coe-Pak) covered with sterile foil	Sterile moist gauze compression, then periodontal dressing (Coe-Pak) covered with sterile foil	3% H₂O₂ foaming test; complete epithelialization defined as absence of foaming
Bastos et al. (2026)	Sutures and an acetate palatal plate	Sutures and an acetate palatal plate	3% H₂O₂ bubble test, graded as absent, partial, or complete
Paz et al. (2025)	Haemostatic collagen sponge	Haemostatic collagen sponge	3% H₂O₂-soaked gauze; complete epithelialization recorded as a binary outcome. Assessed at days 3, 7, 10, 15 and 30
Morshedzadeh et al. (2022)	Palatal acrylic stent; no donor site sutures	Palatal acrylic stent; no donor site sutures (contralateral site in the same patient)	3% H₂O₂; graded as non-, partially, or completely epithelialized
Bitencourt et al. (2022)	Sutures (4 − 0 silk); no stent or dressing reported	Sutures (4 − 0 silk); no stent or dressing reported	Disclosing dye (Mira-2-Ton) with percentage wound closure measured as a continuous variable; no binary definition of complete epithelialization
Isler et al. (2018)	No dressing or stent	No dressing or stent	Re-epithelialization of at least 95% of the wound, assessed both clinically with 3% H₂O₂ over 16 notional sectors and by digital planimetry
Ustaoglu et al. (2017)	Non-eugenol periodontal dressing (Coe-Pak) and an acrylic stent worn for 7 days	Non-eugenol periodontal dressing (Coe-Pak) and an acrylic stent worn for 7 days	3% H₂O₂ bubbling test; complete epithelialization defined as absence of bubbling
Heidari et al. (2017)	Cross mattress silk sutures	Cross mattress silk sutures (contralateral site in the same patient)	3% H₂O₂; complete epithelialization defined as absence of bubbling, incomplete as bubbling
Ozcelik et al. (2016)	No protective material applied	Collagen matrix (Jason-fleece) retained by compressive 5 − 0 sutures	Epithelialization was not an outcome of this trial

*H₂O₂: *hydrogen peroxide. Donor site management applied equally to both arms except in Ozcelik et al., where protective material was applied to the control arm only.

### Risk of bias assessment

**Fig. 2:**
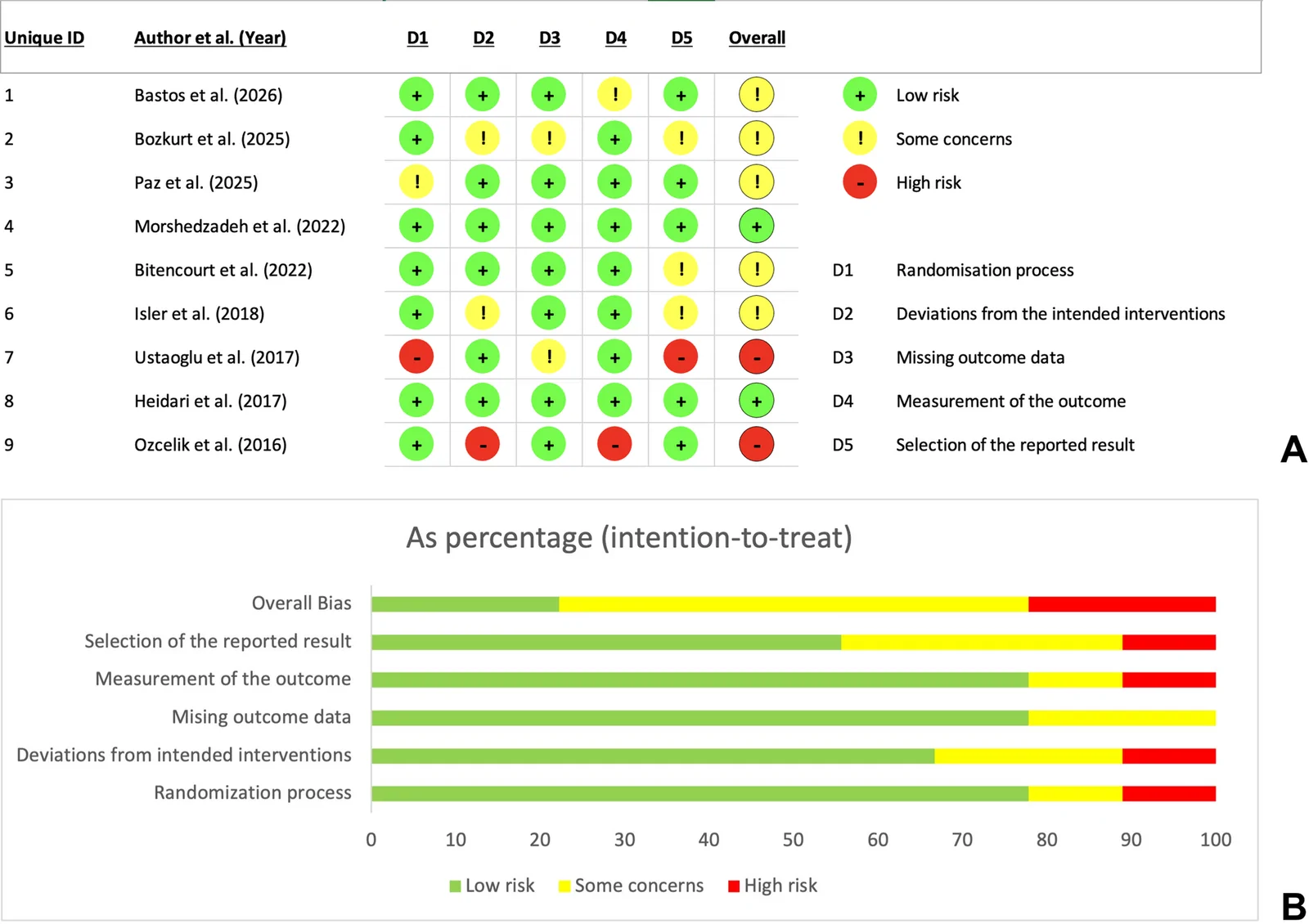
(**A**) RoB-2 risk of bias assessment. (**B**) Collective data (%) for risk of bias, according to RoB-2 domains. RoB-2: risk of bias tool 2

Of the nine included studies, two had a low risk of bias, five were assessed as having some concerns, arising variously from an incompletely described randomization procedure, a per-protocol analysis, unblinded outcome assessment and a reported result that could not be checked against a prespecified analysis, and two studies were considered at high risk of bias owing to unbalanced co-interventions, inadequately concealed allocation, unblinded assessment of a self-reported outcome, and incomplete numerical reporting of prespecified outcomes. Selection of the reported result and the absence of blinding were the most frequent sources of bias across the included studies (Fig. 2).

### Study outcome

#### Postoperative pain by VAS score

**Fig. 3:**
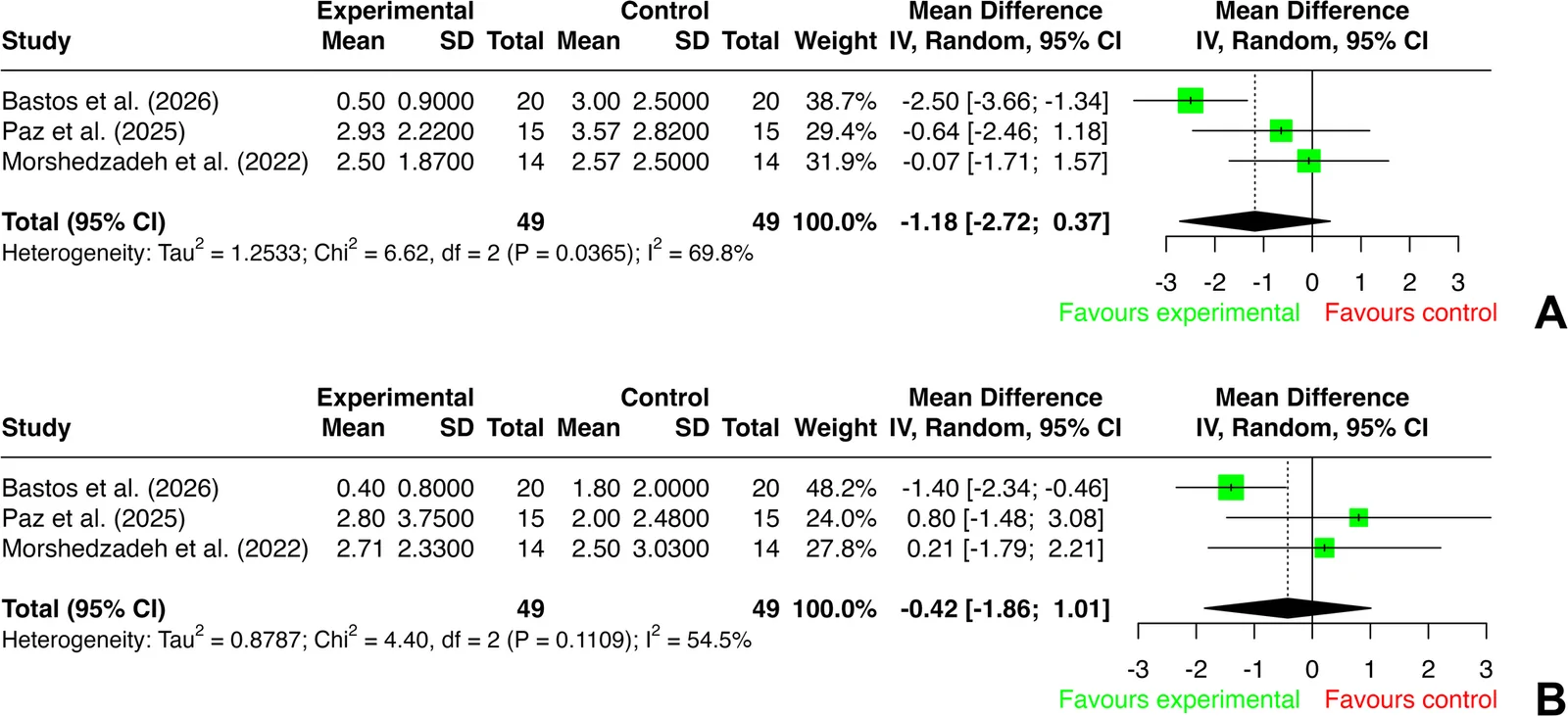
Forest plots of pain (visual analogue scale, VAS) between PBM therapy adjunctive to periodontal surgery and placebo. (**A**) at day 3 post-surgery. (**B**) at day 7 post-surgery. SD: standard deviation, CI: confidence interval. Standard deviations are those reported by the trials, with the split-mouth trial analysed as unpaired

Three of the nine included trials measured donor site pain on a comparable scale. Two of these reported values at day 1; their estimates were inconsistent (MD = -2.60; 95% CI: (-3.54; -1.66); *p* < 0.001 and MD = -0.36; 95% CI: (-1.78; 1.06); *p* = 0.619) and were not pooled [11, 12]. At day 3 the pooled estimate from three trials was not statistically significant (MD = -1.18; 95% CI: (-2.72; 0.37); *p* = 0.135), with moderate heterogeneity (I² = 69.8%, *p* = 0.036). At day 7 the estimate from the same trials was smaller and also non-significant (MD = -0.42; 95% CI: (-1.86; 1.01); *p* = 0.563), with moderate heterogeneity (I² = 54.5%, *p* = 0.111) (Fig. 3A and B) [11–13]. The 95% prediction intervals were (-7.07; 4.71) at day 3 and (-5.54; 4.69) at day 7. In the sensitivity analysis, rescaling the standard deviations of the split-mouth trial for imputed correlations of 0.3 to 0.9 left both estimates non-significant: the pooled mean difference moved from -1.18 to -1.05 at day 3 and from -0.42 to -0.30 at day 7, the weight of that trial rose from 31.9% to 39.6% at day 3 and from 27.8% to 42.2% at day 7, and heterogeneity increased (I² up to 84.8% at day 3 and 75.1% at day 7) [11].

#### Analgesic consumption

Six trials reported analgesic consumption during the first postoperative week, using different medications and reporting units [13–18]. Two reported significantly lower analgesic consumption in the PBM group, at day 3 in one trial and over the first postoperative week in the other; the remaining four reported no between-group difference, one of them without numerical data.

#### Epithelialization

**Fig. 4:**
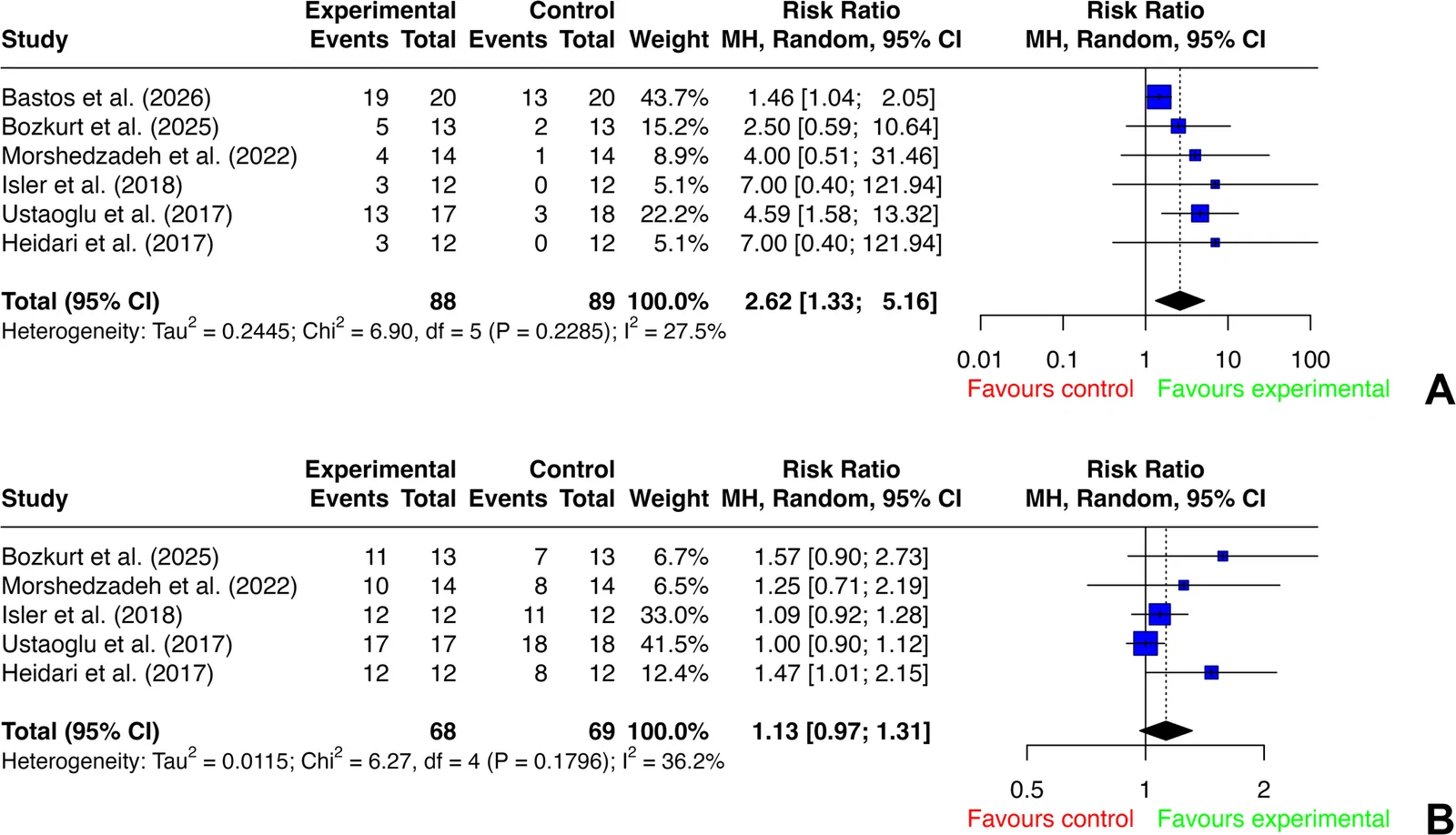
Forest plots of wound complete epithelialization between PBM therapy adjunctive to FGG and placebo. (**A**) at day 14 post-surgery. (**B**) at day 21 post-surgery. CI: confidence interval

Complete epithelialization was reported by six trials at day 14 and by five at day 21. At 14 days the pooled estimate favoured PBM therapy (RR = 2.62; 95% CI: (1.33; 5.16); *p* = 0.005), with low heterogeneity (I² = 27.5%, *p* = 0.229) [11, 12, 15–18]. At 21 days the pooled estimate was not significant (RR = 1.13; 95% CI: (0.97; 1.31); *p* = 0.124), with low heterogeneity (I² = 36.2%, *p* = 0.180) (Fig. 4A and B) [11, 15–18]. In one trial, all donor sites in both groups had healed completely by day 21 [18].

#### Wound remaining area (mm²)

**Fig. 5:**
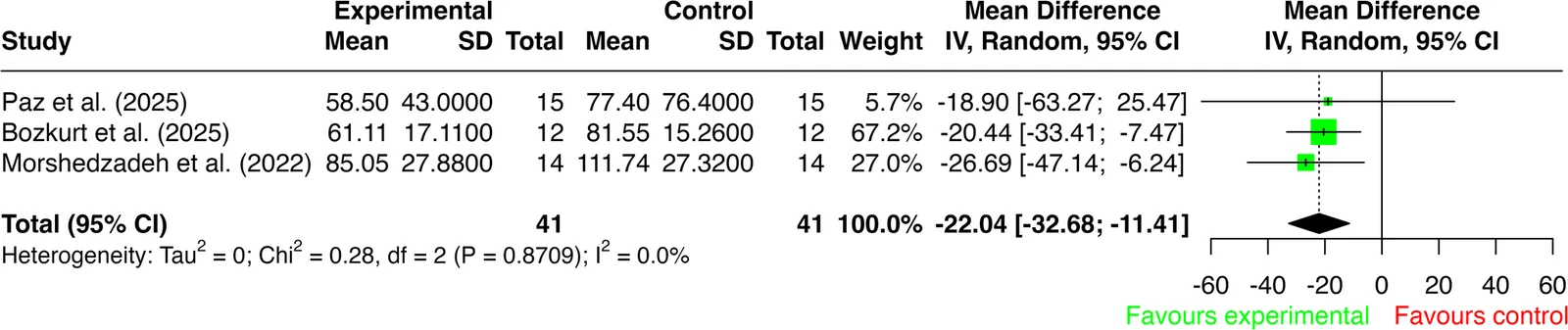
Forest plots of wound remaining area between PBM therapy adjunctive to FGG and placebo at day 7 post-surgery. SD: standard deviation, CI: confidence interval. Standard deviations are those reported by the trials, with the split-mouth trial analysed as unpaired

Three trials reported the mean remaining wound area numerically, of which day 7 was the only time point common to all three [11, 13, 16]. The pooled estimate showed a significant reduction in the PBM group (MD = -22.04 mm²; 95% CI: (-32.68; -11.41); *p* < 0.001), with no heterogeneity (I² = 0%, *p* = 0.871) (Fig. 5). Rescaling the standard deviations of the split-mouth trial for imputed correlations of 0.3 to 0.9 moved the pooled estimate from -22.04 to -25.33 mm², without altering statistical significance or heterogeneity; the weight of that trial rose from 27.0% to at most 78.7%. In a fourth trial the same outcome was measured but presented only graphically [17].

#### Bleeding

Findings on haemostasis were inconsistent. One trial reported significantly less bleeding during the first two postoperative days after PBM therapy than at untreated donor sites [18]. A second trial reported a lower patient-reported bleeding score in the PBM group at 24 h [14]. One trial reported a higher rate of immediate bleeding at laser-treated donor sites on the day of surgery, which the authors attributed to laser-induced vasodilation [11]. In two further trials, immediate bleeding recorded at graft removal did not differ between arms, and isolated early bleeding episodes requiring re-suturing were reported in two test sites and one control site [15, 17].

#### Certainty of evidence

The certainty of the evidence was rated low for complete epithelialization at day 14, downgraded for risk of bias and imprecision, and very low for postoperative pain at day 3, downgraded for risk of bias, inconsistency and imprecision. The domain-by-domain assessment, with the reason for each downgrade, is presented in Table 3.

**Table 3:** Summary of findings: photobiomodulation therapy compared with control for the palatal donor site after graft harvesting

Outcome (No. of participants; studies)	Anticipated absolute effects (95% CI)		Relative effect (95% CI)	Certainty	Reasons for downgrading
	Control	PBM therapy			
Complete epithelialization at day 14 (151 participants; 6 RCTs)	213 per 1000	559 per 1000 (283 to 1000) Difference: 345 more per 1000 (70 more to 787 more)	RR 2.62 (1.33 to 5.16)	⊕⊕◯◯ Low	Risk of bias − 1: only two of the nine included trials were at low risk of bias overall, and 22.2% of the weight came from a trial at high risk. Imprecision − 1: few events and a wide confidence interval. Not downgraded for inconsistency (I² = 27.5%), indirectness, or publication bias.
Postoperative pain (VAS 0–10) at day 3 (84 participants; 3 RCTs)	Mean 2.6 to 3.6 points across control arms	1.18 points lower (2.72 lower to 0.37 higher)	—	⊕◯◯◯ Very low	Risk of bias − 1: as above, and 68.1% of the weight came from trials with some concerns. Inconsistency − 1: I² = 69.8%. Imprecision − 2: the confidence interval includes both no effect and a clinically important reduction. Not downgraded for indirectness or publication bias.

*CI: *confidence interval*; RCT:* randomized controlled trial; *RR: *risk ratio;* VAS*: visual analogue scale*.* Certainty was rated with the GRADE approach across risk of bias, inconsistency, indirectness, imprecision and publication bias; ⊕⊕⊕⊕ high, ⊕⊕⊕◯ moderate, ⊕⊕◯◯ low, ⊕◯◯◯ very low. Absolute effects are calculated from the pooled control-group risk across the contributing trials (19 of 89 donor sites). Participant numbers count patients; in the two split-mouth trials the same patients contribute to both arms, so the arm totals in the forest plots are larger. Publication bias could not be assessed because fewer than ten trials contributed to either analysis

## Discussion

Palatal graft harvesting, despite its established effectiveness in augmenting keratinized gingiva and managing gingival recession, remains associated with substantial donor site morbidity. Although PBM therapy has been proposed as an adjunct to reduce pain and enhance wound healing, the heterogeneity of treatment protocols and the methodological limitations of several included trials preclude definitive conclusions.

### Photobiomodulation and postoperative pain

Pain is recognized as the most prevalent postoperative complication after palatal soft tissue harvesting [5]. While typically acute, some patients recall the discomfort associated with this procedure years later, and rare cases of sensory dysfunction can persist for several months [5, 19, 20]. In the present analysis the two day-1 estimates were inconsistent, at reductions of 2.60 and 0.36 points out of 10, and the pooled reductions of 1.18 points at day 3 and 0.42 points at day 7 did not reach statistical significance. Donor site pain has a characteristic time course: it is greatest during the first three postoperative days, when the acute inflammatory response is most intense, and by the end of the first week has usually fallen close to preoperative levels, a decline that has been linked to stabilization of the clot and the onset of epithelial coverage [19, 21]. The point estimates follow this time course, being largest in the acute phase and diminishing by day 7, but neither pooled estimate reached statistical significance; an analgesic effect can therefore be neither confirmed nor excluded. Moderate heterogeneity and wide prediction intervals at both pooled time points indicate that the effect in a future trial remains largely unpredictable.

Analgesic consumption offers an alternative index of pain but was reported with different medications, dosages, and monitoring durations, and in several trials was not specified at all. Two of the six trials reporting it found significantly lower consumption in the PBM group. Because analgesic use may confound reported pain intensity and is rarely accounted for, VAS-based comparisons must be interpreted with caution [21]. An earlier synthesis of secondary intention gingival wounds could not pool postoperative pain at all, because the assessment methods across trials were too heterogeneous [6]. Here, after restriction to trials measuring pain rather than discomfort on a common scale, three trials remained and neither pooled estimate showed a significant reduction. A synthesis of thirteen trials of PBM therapy across periodontal surgical procedures did report a significant reduction in pain at day 3, together with lower analgesic intake over the first postoperative week [7]. That review was not restricted to palatal donor sites or to a single pain scale, so the discrepancy may reflect the narrower population and the smaller number of contributing trials here rather than a difference in the underlying effect.

An analgesic effect of PBM has been attributed to its action on the peripheral nerve endings of nociceptors, whereby local edema and inflammation decrease, endogenous opioid release increases, and peripheral sensitization is inhibited [22]. These mechanisms derive from laboratory and mechanistic studies rather than from the trials included here and cannot be evaluated from the present clinical data.

The VAS is simple and reliable but is not a purely nociceptive measure: scores are confounded by the patient’s psychological state, particularly dental anxiety and anticipation of pain [23–25]. Patients may also be unable to distinguish pain arising from separate wounds, which introduces measurement bias, particularly in the two split-mouth trials [11, 15].

### Effectiveness of photobiomodulation in the wound healing process

Palatal donor sites heal predominantly through granulation tissue formation, typically requiring up to about four weeks to achieve complete epithelialization, which makes them more susceptible to delayed healing, infection, and fibrous scarring [6, 21]. In the present analysis, PBM therapy more than doubled the proportion of sites achieving complete epithelialization at 14 days and reduced the remaining wound area by approximately 22 mm² at day 7, whereas no significant difference was demonstrable by day 21. The absence of a difference at day 21 reflects a ceiling effect rather than evidence of no effect: by three weeks most sites had healed in both groups, and in one trial every site in both arms had epithelialized completely. Other systematic reviews have reported that PBM therapy accelerates the clinical resolution of oral wounds and yields a higher rate of complete wound epithelialization within the first month compared with untreated controls [5–7, 26, 27]. An earlier synthesis of secondary intention gingival wounds after periodontal surgery reported a similar increase in complete epithelialization (RR = 3.23; 95% CI: (1.66; 6.31)) [6]. That analysis pooled four trials, three of which are also included here, so the present estimate, which is smaller and less precise, is not an independent replication.

PBM therapy is proposed to accelerate tissue repair by targeting mitochondrial cytochrome c oxidase, the primary photoacceptor for red and near-infrared light, thereby elevating ATP synthesis and stimulating growth factors including TGF-β, PDGF, and VEGF, which drive fibroblast proliferation, collagen synthesis, and angiogenesis [6, 7, 22, 28, 29]. These mechanisms provide a rationale for the earlier epithelialization observed at 14 days, but they cannot be evaluated from the clinical outcomes synthesized here.

For the remaining wound area, the three contributing trials followed different assessment schedules, so pooling was possible only at day 7. Their baseline wound sizes also differed, being approximately 90 mm² in one trial and about 134 mm² in the other two. As a proportion of the baseline area still unhealed at day 7 the three trials were comparable: 63% versus 83%, 65% versus 85%, and 46% versus 61% for PBM therapy and control.

The method of assessment also influenced the epithelialization findings. For the assessment of secondary intention healing, the 3% hydrogen peroxide bubbling test serves as the conventional diagnostic standard and detects thin epithelial barriers that clinical photography might miss [17]. Digital planimetry reduces inter-observer error and yields a continuous rather than ordinal variable [17]. In the one included trial that applied both methods, the two methods showed only moderate agreement, which indicates that the choice of assessment method is itself a source of between-study heterogeneity for this outcome [17].

Reported laser parameters varied widely across the included trials, with wavelengths from 660 to 970 nm and energy densities from 1 to 317.99 J/cm². All wavelengths fell within the visible red to near-infrared spectrum (600–1100 nm), consistent with the established optical therapeutic window for effective PBM therapy [30]. Beyond wavelength, fluence, wave mode, irradiance, treatment time, and the number of repetitions all contribute to the biphasic dose response described by the Arndt–Schultz principle, whereby insufficient doses fail to stimulate a response while excessive doses may inhibit it or induce cytotoxicity [22, 30]. In vitro studies of gingival fibroblasts report optimal energy densities in the region of 2.5 to 4 J/cm² for proliferation, whereas higher energy densities appear to be required for analgesia through nerve inhibition or anti-inflammatory pathways [7, 28]. The absence of a standardized dosimetry framework across the included trials therefore constrains any attempt to derive a single optimal protocol from the current evidence.

Patient- and procedure-specific factors also influence postoperative discomfort: graft dimensions and reduced residual palatal thickness are predictors of increased pain, as are procedural complications such as trauma to the healing site or flap necrosis [19, 31–33].

### Bleeding

Postoperative bleeding at the palatal donor site is governed by the vascular anatomy of the region and by the biphasic action of PBM. The donor site, typically located between the canine and the first molar, is perfused by the greater palatine artery and a dense capillary network, making hemostasis a primary clinical concern [5]. PBM influences this vascular bed through the photo-dissociation of nitric oxide from cytochrome c oxidase, triggering vasodilation, which may theoretically counteract immediate hemostasis while simultaneously enhancing platelet procoagulant activity and accelerating clot organization, the latter demonstrated in an animal model of impaired coagulation [22, 34]. The divergent findings across the included trials are consistent with this dichotomy: immediate application may transiently increase bleeding through vasodilation, whereas subsequent sessions likely stabilize the wound bed through accelerated granulation and angiogenesis.

The application of palatal stents, non-eugenol periodontal dressings, and collagen matrices further confounds the assessment of hemostatic outcomes, because these materials actively promote coagulation and provide mechanical compression, obscuring the independent effect of laser irradiation. In one included trial, hemostatic collagen matrices secured by compressive sutures were applied exclusively to the control group while the laser-irradiated sites were left completely exposed, creating an unequal baseline for the comparison of hemostasis [35].

### Strengths and limitations

The strengths of this review are a comprehensive search across five electronic databases, prospective registration with PROSPERO, adherence to the PRISMA statement, verification of all extracted data and citations against the original publications, risk of bias assessment with the Cochrane Risk of Bias 2.0 tool, a random-effects model accommodating heterogeneity in laser parameters, and GRADE rating of the certainty of evidence for the primary outcomes. Pain was pooled only from trials that reported it on a comparable scale.

Several limitations should be considered when interpreting these results. First, the heterogeneity in laser parameters, particularly energy densities ranging from 1 to 317.99 J/cm², precludes the establishment of a universal clinical protocol. Second, internal validity was limited: only two of the nine included trials demonstrated a low risk of bias, while five raised some concerns and two were at high risk, most frequently because the reported result could not be checked against a prespecified analysis or because participants or outcome assessors were not blinded. Third, two included trials used a split-mouth design and did not report the within-patient correlation between paired donor sites. The continuous outcomes were therefore pooled using the standard deviations as reported, which treats the paired observations as independent and overestimates their variance; imputing a correlation of 0.3 to 0.9 and rescaling accordingly did not change the statistical significance of any pooled estimate. For complete epithelialization the paired counts were not reported, so no paired analysis was possible for that outcome. Fourth, the definitions and assessment methods for complete epithelialization differed among the primary studies (Table 2), and one control-group value required for the day-14 estimate was derived from a published figure rather than from reported numerical data. In one trial the participant number differs between outcomes, the source publication reporting 13 per group in its epithelialization table and 12 elsewhere. Fifth, the comparator conditions were not uniform. Donor site management in the control arms ranged from no protective material at all, through sutures alone, to an acetate palatal plate, a palatal acrylic stent, a periodontal dressing, a haemostatic sponge, or a collagen matrix (Table 2); in one trial protective material and compressive sutures were applied to the control arm only, and in that same trial the laser was additionally used to de-epithelialize the graft, so its effect cannot be attributed to PBM therapy alone. Because these measures themselves influence pain perception and wound healing, the control condition was not a single standardized intervention, and part of the between-trial heterogeneity may reflect this variation rather than the laser protocols alone. Sixth, the pain outcome was not measured identically across trials. Two trials scored the visual analogue scale from 0 to 100 and reported pain graphically or not at all, and a third measured discomfort rather than pain [35]; none of the three contributed to the pooled estimates (Table 1). Three trials reported donor site pain on a comparable scale, one of them by postoperative hour, although one of the three did not state the range of the scale explicitly. The remaining three trials used the same 0–10 scale but reported pain only graphically or, in one case, only as within-group comparisons of log-transformed scores, so no between-group means and standard deviations could be extracted. With so few contributing trials, the absence of a demonstrable reduction may reflect limited statistical power as much as absence of effect.

Future research should standardize dosimetry to improve reproducibility. Adoption of photonic fluence and the Einstein unit, which accounts for wavelength-specific photon energy, would improve comparability between red and near-infrared devices. Investigations should also evaluate the impact of flat-top beam profiles and pulsing frequencies on deep-tissue penetration for analgesia, adopt harmonized definitions of complete epithelialization, apply paired-data analysis in split-mouth designs, and report risk-of-bias-relevant procedures transparently. Trials should also stratify patients by systemic factors including smoking status, diabetes, and age, and use molecular biomarkers such as TGF-β1 and VEGF to quantify tissue repair.

## Conclusion

In this meta-analysis of nine randomized controlled trials, PBM therapy may increase the proportion of palatal donor sites achieving complete epithelialization at 14 days, whereas no significant pooled effect was demonstrated on postoperative pain or on epithelialization at day 21. The certainty of evidence was rated low for 14-day epithelialization and very low for postoperative pain, the downgrading being attributable to risk of bias in the primary studies, imprecision of the pooled estimates and, for pain, the small number of trials reporting comparable data. Larger trials with standardized dosimetry and harmonized outcome definitions are needed to establish the clinical value of PBM therapy at this site.

## Supplementary Information

Below is the link to the electronic supplementary material.

Supplementary Material 1 (DOCX 36.9 KB)

## Data Availability

No datasets were generated or analysed during the current study.
