# Supplementary material for: Effects of photobiomodulation therapy on secondary healing of the palatal donor site after free gingival graft harvesting: a systematic review and meta-analysis
Source: Lasers Med Sci. 2026 Nov 7;41(1):287. doi: 10.1007/s10103-026-05073-2 (PMC13743645; doi:10.1007/s10103-026-05073-2)
Supplement: Supplementary file 1 [file 10103_2026_5073_MOESM1_ESM.docx]

**Appendix 1. Search strategies**

Effects of photobiomodulation therapy on secondary healing of the palatal donor site after connective tissue graft harvesting: a systematic review and meta-analysis

Five electronic databases were searched from inception to 20 October 2025. Only reports published in English were considered. No grey literature sources, trial registries or reference lists were searched. The queries are reproduced below exactly as they were run.

**PubMed/MEDLINE**

("free gingival graft"[tiab] OR "FGG"[tiab] OR "connective tissue graft"[tiab] OR "subepithelial connective tissue graft"[tiab] OR "CTG"[tiab] OR "SCTG"[tiab]) AND ("palatal donor site"[tiab] OR "palatal wound"[tiab] OR "palate donor site"[tiab] OR "secondary healing"[tiab] OR "Wound Healing"[mh] OR "second intention"[tiab] OR "palatal wound healing"[tiab] OR "palate wound"[tiab]) AND ("low-level laser therapy"[tiab] OR "Low-Level Light Therapy"[mh] OR "low level laser therapy"[tiab] OR "LLLT"[tiab] OR "photobiomodulation"[tiab] OR "PBM"[tiab] OR "low intensity laser therapy"[tiab] OR "low power laser therapy"[tiab] OR "laser phototherapy"[tiab] OR "diode laser"[tiab] OR "GaAlAs laser"[tiab] OR "laser irradiation"[tiab] OR "laser therapy"[tiab]) AND (“healing” [tiab] OR “recovery”[tiab] OR “wound”[tiab] OR “epithelialization”[tiab] OR “pain”[tiab] OR “discomfort”[tiab] OR “complication”[tiab] OR “analgesic”[tiab] OR “VAS”[tiab] OR “morbidity”[tiab])

**EMBASE**

('free gingival graft':ti,ab,kw OR 'palatal donor site':ti,ab,kw OR 'connective tissue graft':ti,ab,kw OR 'free soft tissue graft':ti,ab,kw OR 'palatal graft':ti,ab,kw OR 'graft harvesting':ti,ab,kw) AND ('photobiomodulation therapy':ti,ab,kw OR 'low level laser':ti,ab,kw OR 'low-level laser':ti,ab,kw OR 'low power laser':ti,ab,kw OR 'low-power laser':ti,ab,kw OR 'low intensity laser':ti,ab,kw OR 'low-intensity laser':ti,ab,kw OR 'LLLT':ti,ab,kw OR 'laser biostimulation':ti,ab,kw OR 'laser therapy':ti,ab,kw OR 'soft laser':ti,ab,kw OR 'cold laser':ti,ab,kw OR 'low-energy laser':ti,ab,kw OR 'therapeutic laser':ti,ab,kw OR 'laser repair':ti,ab,kw) AND (healing:ti,ab,kw OR recovery:ti,ab,kw OR wound:ti,ab,kw OR epithelialization:ti,ab,kw OR 'tissue thickness':ti,ab,kw OR 'color match':ti,ab,kw OR pain:ti,ab,kw OR discomfort:ti,ab,kw OR complication:ti,ab,kw OR analgesic:ti,ab,kw OR VAS:ti,ab,kw OR morbidity:ti,ab,kw)

**Cochrane Central Register of Controlled Trials (CENTRAL)**

(free gingival graft OR palatal donor OR connective tissue graft OR graft harvesting):ti,ab,kw AND (photobiomodulation therapy OR low level laser therapy OR LLLT OR Soft laser OR cold laser OR low-energy laser OR therapeutic laser OR laser biostimulation):ti,ab,kw AND (healing OR recovery OR wound OR epithelialization OR “tissue thickness” OR “color match” OR pain OR discomfort OR complication OR analgesic OR VAS OR morbidity):ti,ab,kw

**SCOPUS**

( TITLE-ABS-KEY ( "free gingival graft" OR "palatal donor site" OR "connective tissue graft" OR "free soft tissue graft" OR "palatal graft" OR "graft harvesting" ) AND TITLE-ABS-KEY ( "photobiomodulation therapy" OR "low level laser" OR "low-level laser" OR "low power laser" OR "low-power laser" OR "low intensity laser" OR "low-intensity laser" OR LLLT OR "laser biostimulation" OR "laser therapy" OR "soft laser" OR "cold laser" OR "low-energy laser" OR "therapeutic laser" OR "laser repair" ) AND TITLE-ABS-KEY ( healing OR recovery OR wound OR epithelialization OR "tissue thickness" OR "color match" OR pain OR discomfort OR complication OR analgesic OR VAS OR morbidity ) ) AND ( LIMIT-TO ( DOCTYPE , "ar" ) )

**Web of Science**

TS=("free gingival graft" OR "palatal donor site" OR "connective tissue graft" OR "free soft tissue graft" OR "palatal graft" OR "graft harvesting") AND TS=("photobiomodulation therapy" OR "low level laser" OR "low-level laser" OR "low power laser" OR "low-power laser" OR "low intensity laser" OR "low-intensity laser" OR "LLLT" OR "laser biostimulation" OR "laser therapy" OR "soft laser" OR "cold laser" OR "low-energy laser" OR "therapeutic laser" OR "laser repair") AND TS=(healing OR recovery OR wound OR epithelialization OR "tissue thickness" OR "color match" OR pain OR discomfort OR complication OR analgesic OR VAS OR morbidity)
